# Delayed diagnosis of selected thoracic and mediastinal conditions in young adults: a focused narrative review of clinical pitfalls, cognitive biases, and system barriers

**DOI:** 10.3389/fmed.2026.1964531

**Published:** 2026-09-25

**Authors:** Ramzi Hazboun, Yaman Ayasa, Asad Omarion, Jana Faroun, Joud Zghyer, Toqa Jawabreh, Yousef Abu Asbeh

**Affiliations:** 1 Al-Quds University Faculty of Medicine, Abu Dis, Palestine; 2 Al Quds Medical Research Association, Jerusalem, Palestine; 3 Al-Quds Bard College, Al-Quds University, Abu Dis, West Bank, Palestine

**Keywords:** airway tumor, cognitive bias, delayed diagnosis, diagnostic pitfalls, esophageal perforation, mediastinal germ cell tumor, thoracic and mediastinal conditions, young adults

## Abstract

**Background:**

Thoracic and mediastinal conditions affecting the esophagus, diaphragm, mediastinum, and airway carry considerable morbidity when diagnosis is delayed. Young adults are often perceived as low risk for serious thoracic disease, and low clinical suspicion, atypical symptoms, and cognitive and healthcare-system barriers can delay recognition.

**Methods:**

This focused narrative review searched PubMed and Scopus, supplemented by Google Scholar, for studies published between January 2000 and May 2026, with selected earlier landmark studies retained where essential. Search terms included “thoracic surgical conditions,” “young adults,” “late diagnosis,” “misdiagnosis,” and “unusual presentation.” Five conditions were selected purposively to illustrate contrasting mechanisms and time-courses of diagnostic delay rather than to catalogue thoracic disease exhaustively. No formal systematic search protocol, study-level quality appraisal, or narrative-review quality instrument was applied.

**Results:**

Five diagnostic categories are examined: esophageal perforation and Boerhaave syndrome, traumatic diaphragmatic injury, primary mediastinal germ cell tumors, airway tumors misdiagnosed as asthma, and Hodgkin lymphoma presenting as an anterior mediastinal mass. Hodgkin lymphoma is included as a mediastinal diagnostic pitfall because it resembles other anterior mediastinal tumors and requires histological sampling for definitive diagnosis. Together, these conditions illustrate how overlapping cognitive and systemic barriers contribute to diagnostic delay. The strength and age relevance of the supporting evidence differ markedly between conditions and are stated explicitly, as much of the foundational literature derives from broader adult populations rather than cohorts restricted to ages 18–40.

**Conclusions:**

Diagnostic delay reflects the combined effects of non-specific presentation, anchoring, premature closure, limited access to imaging, and delayed specialist referral. Consequences differ by condition: in acute mechanical and perforation emergencies, delays measured in hours may affect contamination, deterioration, and operative complexity, whereas in indolent mediastinal malignancies delay principally affects diagnostic complexity, symptom and psychological burden, and time to staging and treatment, while prognosis remains strongly influenced by histological subtype and tumor biology. Structured reassessment after an unexpected treatment response, timely cross-sectional imaging, disease-specific testing, and early specialist referral are practical measures available to first-contact clinicians.

## 1 Introduction

Thoracic surgical disease encompasses a range of life-threatening conditions affecting the lungs, pleura, mediastinum, esophagus, and diaphragm (1). Prompt diagnosis is critical: in conditions such as esophageal perforation, delayed recognition correlates with clinical deterioration, complications, and more complex surgery (2, 3). While these diseases predominantly affect older or high-risk patients, they also occur in young adults, in whom diagnosis may be delayed because of low clinical suspicion.

Young adults, typically defined as those aged 18–40 years, are often perceived by clinicians as being at lower risk for serious thoracic disease. Consequently, symptoms such as chest pain, dyspnea, and gastrointestinal complaints may initially be regarded as benign (4). Atypical presentations further complicate diagnosis, as symptoms in this age group may diverge from classical descriptions (5).

Presentation alone does not explain these delays. Cognitive biases, including anchoring, premature closure, and availability bias, are well-documented contributors to misdiagnosis in emergency medicine and may be especially consequential when serious illness is not initially suspected in young patients (5, 6). Healthcare-system barriers, including limited access to imaging, emergency department overcrowding, and delayed specialist referral, compound this problem, particularly in under-resourced or conflict-affected regions (7).

Diagnostic error is now recognized as a substantial and partly preventable source of patient harm, and the general literature on its causes, burden, and remediation is well developed (8–11). Applied to delayed diagnosis of thoracic and mediastinal disease in young adults, however, that literature remains fragmented: in the course of preparing this review we did not identify a synthesis spanning these conditions in this age group. Most available accounts are disease-specific: they describe delay within a single condition, in populations that are usually not age-restricted, and they do not compare the mechanisms of delay across conditions. What this review adds is a cross-condition synthesis in a single age band, aligning for each condition the clinical presentation, the specific cognitive bias most likely to be operating, the system barrier that prevents correction of the initial impression, and the characteristic timing of delay, and then translating that alignment into the reassessment points available to a first-contact clinician. A second contribution is deliberate evidence transparency: for every condition we state whether the supporting evidence derives from young adults, from mixed-age populations, or from broader adult cohorts extrapolated to this group, so that readers can weigh the cross-condition pattern against the strength of its underlying evidence. We do not claim to be the first review of any individual condition considered here.

This review is a focused, purposive synthesis rather than an exhaustive catalogue of thoracic disease in young adults. The five conditions were not identified by a formal, prespecified selection algorithm; they were chosen because together they span the range of mechanisms and time-courses by which serious thoracic and mediastinal disease is missed in this age group, and because each is well enough described in the literature to support discussion of the reasons for delay rather than merely its occurrence. Specifically, they illustrate five distinct failure modes: an acute mechanical catastrophe whose symptoms imitate common cardiac and gastrointestinal emergencies (esophageal perforation); an injury that is occult at the index presentation and declares itself only after a latent interval of weeks to years (traumatic diaphragmatic injury); a malignancy with a clinically silent growth phase in which a specific, inexpensive test is available but frequently not ordered (primary mediastinal germ cell tumors); a tumor whose symptoms are indistinguishable from the single commonest respiratory diagnosis of young adulthood, so that correct treatment of the wrong disease sustains the error (airway tumors mistaken for asthma); and a malignancy that competes for the same clinical picture as a prevalent infection, so that empirical therapy substitutes for tissue diagnosis (Hodgkin lymphoma mistaken for tuberculosis). Each also differs in the currency of delay: hours in the first, months to years in the second, and weeks to months in the last three (1, 12–25).

Other important thoracic emergencies in young adults, including spontaneous pneumothorax, pulmonary embolism, acute aortic dissection, and acute pericarditis, fall outside this deliberately focused scope. That exclusion is not a judgment of their clinical importance, which is considerable. Spontaneous pneumothorax and acute pericarditis are typically diagnosed at or near first presentation with widely available first-line tests; pulmonary embolism and aortic dissection are supported by validated, extensively studied diagnostic pathways and decision rules and have their own substantial dedicated literature. The conditions selected here share the opposite problem: no validated pathway exists to prompt the clinician, the correct test is often not ordered because the diagnosis is not considered, and the initial error is frequently reinforced rather than corrected by the patient's subsequent course. It is this pattern, rather than thoracic disease in general, that the review addresses.

## 2 Methods

This article is a narrative review of delayed diagnosis of selected thoracic and mediastinal conditions in young adults, focusing on cases with atypical or non-specific symptoms. Unlike a systematic review, its purpose was not to catalogue every existing study but to compile and analyze literature on known diagnostic delays and errors.

The review was guided by the question: “What clinical presentations, cognitive biases, and healthcare-system barriers contribute to delayed diagnosis of selected thoracic and mediastinal conditions in adults aged 18–40 years?” This question framed the search strategy, article selection, and synthesis, with attention to atypical presentations, diagnostic reasoning pitfalls, and structural healthcare barriers.

A literature search was conducted in PubMed and Scopus, supplemented by Google Scholar for wider coverage of case reports and grey material, for studies published between January 2000 and May 2026; landmark studies published before 2000 were retained where essential for historical context or foundational evidence. Free-text keywords, used individually and in combination, included “thoracic surgical problems,” “young adults,” “delayed diagnosis,” “misdiagnosis,” “diagnostic delay,” and “atypical presentation.” These databases were selected because the review question is clinical and diagnostic: PubMed and Scopus provide direct coverage of the biomedical, surgical, and health-services literature in which studies of diagnostic delay, diagnostic error, and clinical presentation are indexed. The review does not address chemical synthesis, drug development, or the engineering of instruments or techniques, and chemistry- and patent-oriented databases were therefore not searched.

Articles were included based on relevance to the review question, clinical significance, and information on delayed diagnosis, unusual presentation, or diagnostic pitfalls in young adults. Systematic reviews, meta-analyses, and larger cohorts or case series were prioritized; case studies were selected only when they demonstrated specific diagnostic pitfalls and are labeled accordingly. Where age-specific data were unavailable, general adult-population studies were used if relevant to young adults.

Articles were excluded if unrelated to the reviewed conditions, if they were animal studies, were not written in English, or did not address diagnostic delay, atypical presentation, or diagnostic pitfalls relevant to the review question.

No formal study-level quality appraisal was performed, and no narrative-review quality instrument was used. In particular, the Scale for the Quality Assessment of Narrative Review Articles (SANRA) was not applied at any stage: it did not guide the search, the selection of studies, the assessment of study quality, or the structure of this review, either during its original conduct or during revision. Readers should therefore interpret the synthesis presented here as a purposive expert reading of the literature, with the limitations set out in Section 4.7, and not as a quality-appraised or systematically screened evidence review.

During revision, each citation supporting a substantive claim was re-examined to record its study design and the age composition of its study population. Sources are described in the text, and summarized in Table 1, in one of three ways: evidence drawn from young adults or from populations concentrated in the 18–40 range; evidence from mixed-age adult populations that include young adults but are not age-stratified; and evidence from broader adult populations, most commonly older than this review's target group, which is used cautiously and identified explicitly wherever it underpins a claim. Where the age composition of a source could not be established, that uncertainty is stated rather than resolved by assumption. This labelling was performed at the revision stage as an act of reporting transparency; it is not a quality-appraisal procedure and does not constitute a risk-of-bias assessment.

**Table 1:** Diagnostic pitfalls, contributing factors, consequences, and evidence base for delayed diagnosis across the five reviewed thoracic and mediastinal conditions.

Condition	Main diagnostic pitfall	Key factors contributing to delay	Principal consequences of delay	Evidence base and population relevance
Esophageal perforation/Boerhaave syndrome	Misattributed to MI, peptic ulcer disease, PE, or aortic dissection	Clinical: non-specific chest/epigastric pain, dyspnea, subcutaneous emphysema Cognitive: anchoring on an initial cardiac or GI diagnosis System: clinician unfamiliarity; delayed CT/fluoroscopic confirmation	Acute (hours matter): diagnosis beyond 24 h linked to substantially higher mortality; greater contamination and surgical complexity (2,3,8–11)	Systematic review (2); retrospective cohort (3) and individual-patient-data meta-analysis (8); retrospective imaging study (10); narrative decision guidance (11); one case report (9). Population: broader adult — the key cohort has a median age in the seventh decade (3), and the only source within 18–40 is a single case report (9). Extrapolate to young adults with caution.
Traumatic diaphragmatic injury	Occult at initial trauma evaluation	Clinical: tamponade effect of herniated organs; masking by concurrent injuries; no pathognomonic early signs Cognitive: low suspicion amid more apparent injuries System: missed CT signs (collar, dependent viscera); delayed follow-up imaging	Latent then acute (weeks to years, then hours): delayed herniation with bowel obstruction or strangulation requiring urgent surgery (1,12–16)	Narrative review (1); systematic review with diagnostic-accuracy meta-analysis (12); imaging review (13); practice management guideline (14); two case series (15,16). Population: mixed-age general trauma — not age-stratified, but mechanisms of major blunt trauma are concentrated in younger adults. Applicability to 18–40 is plausible but not demonstrated.
Primary mediastinal germ cell tumors (PMGCTs)	Failure to obtain tumor markers (AFP, *β*-HCG, LDH) before treating a presumed alternative diagnosis	Clinical: insidious growth; compressive symptoms appear late Cognitive: anchoring on thymoma/lymphoma without marker integration System: inconsistent marker testing; imaging without histopathological correlation	Indolent (weeks to months): delayed tissue diagnosis and staging; late-stage presentation, especially non-seminomatous subtypes; prognosis strongly influenced by histology and tumor biology (17–21,38)	Literature and pathology reviews (17–19,38); large pathological series, *n* = 322 (20); radiologic–pathologic correlation (21); retrospective age- and sex-stratified cohort (22). Population: young adult — the disease itself is concentrated in men aged approximately 20–39, so this section requires least extrapolation. No cohort quantifies delay or its outcome effect.
Airway tumors (tracheal and endobronchial)	Misdiagnosed as asthma, COPD, or bronchitis	Clinical: progressive dyspnea, wheeze, stridor, recurrent infections Cognitive: availability bias favoring asthma System: delayed bronchoscopy; unremarkable initial radiographs; referral-center bias	Indolent then acute (months): ∼12-month mean latency to referral in referred series; risk of airway compromise/emergency admission (23–27)	Narrative reviews (23,24,26); national retrospective analysis (25); one case report in a 39-year-old man (27). Population: broader adult — tracheal malignancy predominantly affects older adults, and reported latencies are not age-specific. Relevance to 18–40 is mechanistic (asthma is a more available competing diagnosis in the young), not epidemiological.
Hodgkin lymphoma (mediastinal diagnostic pitfall)	Misattributed to tuberculosis or other infection; empirical therapy substitutes for tissue diagnosis	Clinical: B symptoms (fever, night sweats, weight loss) overlapping with TB Cognitive: anchoring on TB despite absent microbiological confirmation System: empirical anti-TB therapy before histological confirmation; limited biopsy access; low diagnostic yield of fine-needle aspiration	Indolent (weeks to months): delayed tissue diagnosis and treatment initiation; prolonged symptom and psychological burden; associated with later stage at diagnosis in one cohort (33). The prognostic impact of delay remains uncertain and is strongly influenced by histological subtype and tumor biology; two cohorts found no association between delay and survival (28–33,39)	Guideline (28); adolescent and young adult review (39); two retrospective cohorts (*n* = 50, 31; *n* = 163 with median age 48, 33); three case reports (29,30,32). Population: mixed — disease epidemiology is age-specific (among the most common cancers of ages 15–39, 39), but the delay-specific cohorts are mixed-age adult. Case reports describe patients aged 18–40.

AFP, alpha-fetoprotein; β-HCG, beta-human chorionic gonadotropin; COPD, chronic obstructive pulmonary disease; CT, computed tomography; GI, gastrointestinal; LDH, lactate dehydrogenase; MI, myocardial infarction; PE, pulmonary embolism; TB, tuberculosis. Descriptors in the evidence column indicate study design and the age composition of the study population as reported in the source; they are reporting labels intended to help readers weigh each row and do not constitute a formal quality appraisal, risk-of-bias assessment, or level-of-evidence grading.

## 3 Results

The following subsections (3.1–3.5) present five diagnostic categories in which delayed diagnosis has been reported in young adults. Section 3.6 synthesizes the cognitive biases and healthcare-system barriers common to them.

### 3.1 Esophageal perforation and Boerhaave syndrome

Esophageal perforation is uncommon but serious (2, 3). Boerhaave syndrome, spontaneous transmural perforation from acutely elevated intra-esophageal pressure typically occurring after vomiting, is among the most difficult diagnostic challenges (2). Mortality varies by series and is influenced by diagnostic delay, degree of contamination, and comorbidities (3, 26).

The evidence linking timing to outcome in this condition is strong but is not derived from young adults. Shaker et al. found that mortality was higher when diagnosis occurred more than 24 h after symptom onset, establishing diagnostic timing as a significant clinical parameter; the median age in that cohort was in the seventh decade (3). Vermeulen et al. reached concordant conclusions in an individual-patient-data meta-analysis spanning a similarly broad adult age range (26). This evidence establishes that esophageal perforation is a time-critical diagnosis in adults generally. It should not be read as an age-specific estimate of risk or of the effect of delay in adults aged 18–40, for whom no comparable cohort data were identified; the mechanism of injury and the physiology of mediastinal contamination, however, give no reason to expect that timeliness matters less in younger patients, whose lower comorbidity burden may instead mask deterioration until it is advanced.

Diagnostic difficulty arises from a diverse symptomatology: chest pain, epigastric pain, dyspnea, and subcutaneous emphysema can mimic myocardial infarction, peptic ulcer disease, pulmonary embolism, or aortic dissection (2, 27). Duehring and Rubin described a healthy young adult whose Boerhaave syndrome was initially misdiagnosed as upper gastrointestinal disease; this is a single case report, illustrative of the pattern rather than generalizable, and is the only source in this section that reports a patient within the review's age band (27).

A systematic review by Sdralis et al. found that diagnostic delay beyond 24 h remains common, largely due to nonspecific symptoms and unfamiliarity with the condition (2). Wei et al. found that CT with oral contrast can be performed initially in suspected esophageal perforation and may obviate fluoroscopy (28). Søreide and Viste's published decision-making guidelines emphasize integrating these clinical, radiologic, and temporal factors (12).

For the outpatient or first-contact clinician the operative trigger is the sequence rather than the symptom: severe chest or epigastric pain beginning immediately after forceful vomiting or retching warrants emergency assessment rather than symptomatic treatment and outpatient review, particularly when accompanied by dyspnea, subcutaneous emphysema, or systemic upset. Because early cross-sectional imaging changes management and is time-critical, this presentation is one in which same-day emergency referral, rather than an outpatient imaging request, is the appropriate escalation (2, 12, 28).

### 3.2 Traumatic diaphragmatic injury and delayed diaphragmatic hernia

Diaphragmatic injury from blunt or penetrating thoracoabdominal trauma is well recognized yet often missed clinically (1). Reported incidence varies by injury mechanism, diagnostic method, and population, but undetected injury appears common regardless of exact figures (29). When unrecognized initially, diaphragmatic hernia can develop weeks to years later, presenting emergently with obstruction or strangulation of herniated abdominal contents (1, 13, 14).

Rashid et al. described anatomical and physiological factors that obscure early recognition, including the tamponade effect of herniated organs, masking by concurrent injuries, and the absence of pathognomonic early symptoms (1). In young adults, motor vehicle collisions, sports injuries, and workplace trauma are common causes of missed diaphragmatic injury (1, 29).

CT is essential but imperfect for diagnosis (29–31). Panda et al. described CT signs, including the collar sign, the dependent viscera sign, and diaphragmatic discontinuity, that reduce under-diagnosis (30). The systematic review and diagnostic-accuracy meta-analysis by Reitano et al. found moderate-to-good CT performance in blunt trauma, though subtle defects may still be missed (29). The Eastern Association for the Surgery of Trauma practice management guideline addresses evaluation and management of this injury pattern (31). The evidence in this section derives from general trauma populations rather than from age-restricted cohorts. Such populations typically contain a substantial proportion of young adults, because the dominant mechanisms of major blunt trauma are concentrated in younger age groups, but the studies cited are not stratified by age and do not report age-specific diagnostic accuracy. Their findings are therefore best read as mixed-age adult evidence that plausibly, but not demonstrably, applies to adults aged 18–40.

Delayed diaphragmatic hernia often presents very differently from the inciting trauma (1, 13, 14). Zhao et al., reporting 40 cases of delayed traumatic diaphragmatic rupture, observed predominantly gastrointestinal symptoms including abdominal pain, nausea, vomiting, and obstruction, sometimes occurring years after an injury and with no clearly identifiable precipitant (13). Lu et al. reported comparable findings in a case series requiring surgical repair after delayed presentation (14). Both are uncontrolled case series and cannot support estimates of frequency or of the population-level risk of delay.

The practical implication for outpatient practice is a history-taking one: in a patient presenting with unexplained gastrointestinal, respiratory, or chest symptoms, a history of previous thoracoabdominal trauma remains relevant long after the event, and its presence should lower the threshold for cross-sectional imaging rather than being discounted because the injury was assessed and cleared at the time. This is a reassessment prompt, not a screening recommendation: no evidence identified supports routine imaging of asymptomatic patients after thoracoabdominal trauma (1, 13, 14, 31).

### 3.3 Primary mediastinal germ cell tumors

Primary mediastinal germ cell tumors (PMGCTs) predominantly affect young men aged 25–35 years and account for roughly 15%–20% of anterior mediastinal masses in this group, presumably arising from primitive germ cells retained in the mediastinum during embryogenesis (32). Non-seminomatous PMGCTs carry a worse prognosis than their testicular counterparts, which is one reason prompt recognition is emphasized, although the independent effect of diagnostic delay on outcome has not been quantified in this group (32, 33).

This is the one condition in this review for which the evidence base is intrinsically concentrated in the review's target age band: the disease itself occurs predominantly in young adult men, and the descriptive, pathological, and radiological literature cited here therefore describes populations largely within or adjacent to the 18–40 range (32–35). Conclusions in this section consequently require less extrapolation than elsewhere in the review. The literature is nevertheless descriptive rather than comparative: it consists of pathological series, radiological correlation studies, and narrative reviews, and no cohort study was identified that quantifies the frequency of diagnostic delay in PMGCT or measures its effect on outcome.

Serum tumor markers are a routine component of the evaluation of a young man with an anterior mediastinal mass: elevated AFP and *β*-HCG strongly suggest PMGCT, while LDH provides additional but non-specific prognostic information. Failure to obtain these markers may delay recognition and histopathological confirmation. Fichtner et al. noted that markedly elevated AFP suggests a yolk sac tumor, and that elevated *β*-HCG supports the diagnosis in the appropriate clinical context (33, 36).

PMGCTs often follow an insidious course, growing in the mediastinum before symptoms (dyspnea, cough, chest pain, superior vena cava syndrome, or dysphagia) emerge from mass effect (32). Large series show PMGCTs are often sizeable at diagnosis, consistent with a clinically silent phase, though size alone does not confirm delayed presentation; prognosis is also shaped by histological subtype and tumor biology (15, 32, 34).

Imaging overlap between PMGCTs, thymoma, and lymphoma is a further source of confusion. Azour et al. showed that accurate distinction requires integrating imaging with clinical demographics, tumor markers, and histopathology (15), and Nam et al. found that the spectrum of anterior mediastinal masses varies significantly by age and sex, which is directly relevant to a young-adult population (37). Ghigna and Thomas de Montpreville reach a convergent conclusion from the pathologist's perspective, arguing that mediastinal lesions should be approached by compartment of origin with age, sex, imaging, and laboratory findings integrated before a diagnosis is settled (35). Together these sources support obtaining tumor markers early in young men with anterior mediastinal masses, and before treatment is directed at a presumed alternative diagnosis (33, 35, 36).

The corresponding practical point is one of sequence rather than of obligation: in a young man with an anterior mediastinal mass, AFP, *β*-HCG, and LDH are inexpensive, widely available, and informative, and are best obtained before empirical treatment or definitive-intent therapy is directed at a presumed thymoma, lymphoma, or infection. We deliberately do not describe marker testing as mandatory, since no identified guideline or trial establishes it as an obligatory precondition of treatment; the defensible statement is that it is a standard component of evaluation whose omission is a recognized and avoidable source of delayed recognition.

### 3.4 Airway tumors: tracheal and endobronchial lesions

Tracheal and endobronchial tumors are an under-recognized source of diagnostic delay because their symptoms, progressive dyspnea, wheeze, stridor, and recurrent airway infections, closely resemble asthma, COPD, or recurrent bronchitis, a problem compounded in young adults, in whom asthma is common (16, 17).

Madariaga and Gaissert reported that misdiagnosis as asthma or COPD is a major cause of delay in malignant tracheal tumors, with a mean latency to thoracic surgical referral of approximately 12 months, an estimate that may be affected by referral-center selection bias (17). Stevic and Milenkovic similarly found that non-specific symptoms and unremarkable chest radiographs delay the diagnosis of tracheobronchial tumors (16).

A nationwide analysis by Nouraei et al. found substantial delay between presentation and diagnosis of primary tracheal carcinoma, with some cases identified only during an airway-compromise emergency, although preventive measures were not directly evaluated (18). Sherani et al. likewise emphasized the diagnostic challenge and the role of bronchoscopy (19). The delay estimates in this section derive from adult populations that are not age-restricted, and primary tracheal malignancy is predominantly a disease of middle and later life; the reported latencies should therefore be read as describing the pattern of misattribution to obstructive airway disease in adults generally, not as measured delay intervals in patients aged 18–40. Their relevance to young adults is mechanistic rather than epidemiological: the competing diagnosis of asthma is more readily available in a young patient, not less, so the reasoning failure the literature describes is at least as likely in this age group even though the tumors themselves are rarer. The only source in this section reporting a patient within the review's age band is a single case report (20).

Bronchial carcinoid is a notable pitfall in younger patients. Emeryk et al. described a 39-year-old man treated for asthma for approximately 10–11 months, with persistent wheeze, hemoptysis, and cough unresponsive to inhaled beta-2 agonists and corticosteroids, in whom bronchoscopy ultimately revealed an occlusive endobronchial carcinoid; the authors concluded that patients treated unsuccessfully for asthma or COPD should undergo bronchoscopic evaluation (20). As a single case report this illustrates the pattern without quantifying it.

Several features should prompt reconsideration of an asthma diagnosis rather than escalation of asthma therapy: wheeze that is persistent, monophonic, or focal rather than diffuse and variable; stridor; hemoptysis; recurrent pneumonia in the same anatomical distribution; a flow-volume loop suggesting fixed or extrathoracic obstruction; and failure to obtain the expected response to appropriate, correctly administered therapy (16, 18–20). The last of these is the most useful and the least specific, and it is worth stating precisely what can and cannot be recommended about its timing. Contemporary primary-care asthma guidance advises that where symptoms persist, are more typical of an alternative diagnosis, or fail to benefit from controller therapy, the diagnosis should be reviewed and alternative causes considered, and it uses a trial of approximately four weeks of anti-inflammatory treatment as one benchmark for confirming or questioning the diagnosis, with referral recommended where the diagnosis is difficult or symptoms remain uncontrolled despite adequate therapy, good adherence, and correct inhaler technique (38). That provides a defensible, guideline-anchored review point at around four weeks after starting or stepping up treatment. It does not establish a validated interval after which airway tumors must be excluded, and no such threshold was identified; the trigger for imaging and, where indicated, bronchoscopy is the absence of an expected response together with one or more of the features listed above, rather than the passage of a fixed period alone (16, 19, 20, 38). Chest CT is warranted in patients with persistent wheeze, stridor, or dyspnea despite standard therapy, in order to exclude underlying tracheobronchial pathology (16, 19, 20).

### 3.5 Hodgkin lymphoma presenting as an anterior mediastinal mass

Hodgkin lymphoma (HL) is included here not as a thoracic surgical disease but as a mediastinal diagnostic pitfall, since it commonly presents as an anterior mediastinal mass resembling PMGCT, thymoma, and other mediastinal tumors, and its confirmation typically requires thoracic surgical or image-guided biopsy even though subsequent management is hemato-oncological (39). HL is among the more common malignancies of early adulthood: Kahn and Kelly describe it as one of the most common cancers in the adolescent and young adult population, which they define as ages 15–39, and note that survival gains in this group have lagged behind those achieved in other age groups (40). This is one of the few sources in the review whose defining population corresponds closely to the 18–40 band, and it supports the epidemiological premise of this section: a mediastinal mass with constitutional symptoms in a young adult is a presentation in which lymphoma is genuinely likely, not merely possible.

The evidence on delay in HL, by contrast, comes from mixed-age adult cohorts. Norum reported a median diagnostic delay of four months (range 0–48 months) in a retrospective series of 50 patients, with the longest delays in the lymphocyte-predominant subtype; delay showed no significant association with stage, relapse rate, or death, and the author concluded that diagnostic delay did not appear to influence stage distribution, relapse rate, or short-term survival in that cohort (23).

Antel et al. retrospectively studied 163 consecutive patients with aggressive non-Hodgkin lymphoma or HL in a tuberculosis- and HIV-endemic setting, with a median age of 48 years (range 15–86). The median interval between first healthcare contact and diagnostic biopsy, which the authors term the healthcare practitioner interval and which was the longest single component of the pathway, was seven weeks. Using a threshold of more than six weeks to define diagnostic delay, multivariable analysis found delay to be associated with late-stage disease and with a diagnosis of HL rather than non-Hodgkin lymphoma; the median healthcare practitioner interval was 12 weeks for HL compared with 6 weeks for non-Hodgkin lymphoma. Patients receiving tuberculosis therapy had a median interval four weeks longer, a difference that did not reach statistical significance in this sample; fine-needle aspiration cytology was diagnostic for lymphoma in only 11% of cases and was itself associated with longer time to diagnosis. Diagnostic delay was not associated with overall survival. Notably, the authors attributed the longer interval in HL in part to a younger median age in whom a cancer diagnosis is less likely to be suspected, an interpretation that directly supports the age-related risk-underestimation mechanism proposed in this review, though it remains the authors' inference rather than a tested hypothesis (25).

Case-level reports illustrate the mechanism. Banerjee et al. described a 23-year-old with an anterior mediastinal mass treated as tuberculosis despite negative AFB cultures, with HL confirmed four months later; Putra et al. reported two young adults treated for tuberculous lymphadenitis before immunohistochemistry confirmed HL; and Sawaftah et al. described a young woman with chronic respiratory symptoms in whom HL was eventually diagnosed (21, 22, 24). These are case reports and case pairs: they demonstrate that the error occurs and how it unfolds, but they cannot indicate how often it occurs.

Taken together, this literature supports obtaining tissue for diagnosis in a young adult with a mediastinal or nodal mass and B symptoms rather than allowing empirical anti-infective therapy to substitute for a tissue diagnosis, particularly where microbiological confirmation of tuberculosis is absent. Where fine-needle aspiration is used as the first sampling step, its limited yield in lymphoma should be anticipated, and a non-diagnostic aspirate should prompt progression to excisional or core biopsy rather than repetition (21, 24, 25). The prognostic consequence of delay in HL specifically remains uncertain and is discussed in Section 4.5.

### 3.6 Cognitive biases and healthcare-system barriers

Diagnostic delay in these conditions is not attributable to clinical presentation alone; it is compounded by cognitive error and structural healthcare barriers that operate across diagnostic contexts, and particularly when clinicians evaluate young adults in whom serious pathology is not the leading consideration. Diagnostic error is estimated to cause serious harm to large numbers of patients annually, which underlines the public health relevance of the pitfalls reviewed here (8).

Anchoring bias, fixation on an initial hypothesis despite new clinical data, is among the most commonly reported cognitive errors in the diagnostic-error literature (6). In young adults, the initial framework often centers on benign conditions and may persist despite an evolving presentation, as seen in the Boerhaave syndrome case (Section 3.1) initially attributed to gastrointestinal disease, and the Hodgkin lymphoma cases (Section 3.5) initially attributed to tuberculosis. Hartigan et al. observed that once a diagnostic label is applied in emergency medicine, it tends to persist through handovers and subsequent encounters (6).

Premature closure, accepting a diagnosis before adequate verification, often under time pressure or fatigue, is closely related to anchoring and was identified by Newman-Toker et al. as one of the most common cognitive failure modes in emergency department diagnostic error (5, 10).

Availability bias, whereby more familiar diagnoses are disproportionately weighted, may further disadvantage young adults with rare thoracic conditions (5). The airway tumor cases (Section 3.4), where persistent wheeze was repeatedly treated as asthma before bronchoscopy revealed an endobronchial tumor, illustrate this: asthma is a readily available diagnosis for wheeze, while an endobronchial tumor is rarely considered until standard treatment fails.

At the system level, Morley et al. found in the general emergency department population that overcrowding is independently associated with delayed assessment and adverse outcomes, though causal inference is complex and its specific relevance to young adults with atypical thoracic presentations remains unquantified (7). Additional barriers include limited after-hours imaging access, delayed subspecialty consultation, poor primary-to-secondary care communication, and, in low-resource settings, absent diagnostic infrastructure. Wright et al. identified structured diagnostic checklists, improved handover protocols, and cognitive-bias education as evidence-supported interventions, though their applicability to young adults with undifferentiated thoracic symptoms warrants prospective study (9).

## 4 Comment

### 4.1 Overview of findings

This review examined delayed diagnosis across five thoracic and mediastinal diagnostic categories in adults aged 18–40 years. Despite differing in epidemiology, urgency, prognosis, and management, these conditions share a common pattern: serious disease may be considered late when the patient is young and the initial presentation is non-specific.

These findings address the review question directly: diagnostic delay across the reviewed conditions arises from the interaction of three overlapping domains, namely clinical, cognitive, and system-related factors. The disease-specific findings above illustrate these domains in practice; the following sections synthesize them thematically.

### 4.2 Clinical presentations contributing to delay

Clinical presentation contributed to delay in all five conditions through a single shared mechanism: the presenting symptoms are the common symptoms of common diseases. Chest pain, dyspnea, cough, wheeze, epigastric pain, fever, weight loss, dysphagia, and gastrointestinal complaints overlap extensively with benign gastrointestinal disease, asthma, respiratory infection, tuberculosis, musculoskeletal pain, and anxiety, all of which are far more prevalent in young adults than any condition reviewed here. What distinguishes the five conditions is not the symptom but the pattern: symptoms that are focal rather than diffuse, that are persistent or progressive rather than episodic, that recur in the same anatomical distribution, that follow a specific precipitating event, or that fail to respond to treatment that should work. The disease-specific expressions of this pattern are set out in Sections 3.1–3.5 and summarized in Table 1; the corresponding reassessment points are consolidated in Table 2 rather than repeated here.

**Table 2:** Red flags and reassessment points for first-contact clinicians assessing thoracic symptoms in adults aged 18–40.

Presentation prompting reconsideration	What should prompt a change of course	Reasonable next step	Basis and strength
Severe chest or epigastric pain beginning immediately after forceful vomiting or retching, particularly with dyspnea, subcutaneous emphysema, or systemic upset	The temporal sequence itself; do not treat symptomatically and review later	Same-day emergency assessment; CT with oral contrast is an appropriate first study where perforation is suspected	Systematic review and cohort evidence in broader adult populations; direction well supported, magnitude in 18–40 not established (2,3,8,10,11)
Wheeze that is persistent, monophonic, or focal; stridor; hemoptysis; recurrent pneumonia in the same anatomical distribution; flow-volume loop suggesting fixed obstruction	Absence of the expected response to appropriate, correctly administered and adhered-to therapy — review the diagnosis rather than escalating treatment	Review the basis of the asthma diagnosis; chest imaging (CT where symptoms persist despite standard therapy); bronchoscopy where indicated; specialist referral where the diagnosis is difficult	Guideline-anchored for the reassessment step (41); tumor-specific evidence is review-level and case-level (23,25–27). No validated time threshold exists; a review point at approximately 4 weeks after starting or stepping up treatment follows existing asthma guidance, not a rule for excluding tumor
Anterior mediastinal mass in a young man	Before treatment is directed at a presumed thymoma, lymphoma, or infection	AFP, β-HCG, and LDH; specialist referral; imaging interpreted together with demographics, markers, and histology	Standard component of evaluation per reviews and pathological literature; not framed as mandatory by any identified guideline (17–19,21,38)
Mediastinal or nodal mass with B symptoms, especially where tuberculosis is treated empirically without microbiological confirmation	Absence of microbiological confirmation, or failure to respond to anti-tuberculosis therapy; a non-diagnostic fine-needle aspirate	Tissue diagnosis (excisional or core biopsy) rather than repeated aspiration or continued empirical therapy	Retrospective cohort (33) and case reports (29,30,32); guideline for diagnostic requirement (28)
Unexplained gastrointestinal, chest, or respiratory symptoms in a patient with previous thoracoabdominal trauma, however remote	The trauma history remains relevant even if the patient was assessed and cleared at the time	Cross-sectional imaging with occult diaphragmatic injury explicitly in the differential	Narrative review, guideline, and case series; a reassessment prompt, not a basis for routine imaging of asymptomatic patients (1,13–16)

Table 2 is a prompt for diagnostic reconsideration, not a screening protocol or a validated decision rule. Recommendations are proportional to the evidence described in Section 3; where no threshold is supported by evidence, none is proposed.

### 4.3 Cognitive biases contributing to delay

Cognitive factors compound these clinical difficulties. Anchoring bias occurs when clinicians remain fixed on an initial diagnosis, such as asthma, tuberculosis, gastrointestinal disease, or minor trauma, despite persistent or atypical symptoms, and premature closure occurs when a common diagnosis is accepted before serious alternatives are excluded (5, 6). Availability bias is especially relevant in young adults, since common diagnoses (asthma, tuberculosis, benign gastrointestinal disease) are more readily considered than rarer thoracic or mediastinal disease; these patterns reflect predictable diagnostic vulnerabilities rather than individual negligence (5, 6, 10, 11).

Age-based risk underestimation is a cross-cutting issue: young adults may be perceived as being at low risk of malignancy or severe pathology, which delays escalation to imaging, bronchoscopy, tumor-marker testing, specialist referral, or biopsy, and is particularly important when symptoms recur, fail to respond to therapy, or present with red flags such as hemoptysis, stridor, superior vena cava symptoms, unexplained weight loss, or abnormal imaging. This mechanism is not merely inferred from the cognitive-error literature. It is also offered by clinical investigators as an explanation of their own data: Antel et al. attributed part of the longer diagnostic interval observed in HL to the younger median age of those patients, in whom a cancer diagnosis is less likely to be suspected (25). It is reinforced structurally as well as cognitively, since several widely used suspected-cancer referral pathways are explicitly age-thresholded and do not apply below the age of 40, a point developed in Section 4.6 (41).

### 4.4 Healthcare-system barriers contributing to delay

Healthcare-system barriers reinforce clinical uncertainty and cognitive bias: delayed access to imaging, limited bronchoscopy availability, delayed specialist referral, inadequate follow-up, and delays in tissue diagnosis, compounded by emergency department crowding and time pressure (7, 9). These barriers matter because each condition depends on a specific diagnostic step: CT with oral contrast or fluoroscopic esophagography for suspected perforation (12, 28); careful CT interpretation, with follow-up imaging where needed, for diaphragmatic injury (29–31); AFP, *β*-HCG, and LDH testing for anterior mediastinal masses (33, 35, 36); bronchoscopy for persistent airway symptoms (16, 19, 20); and timely tissue diagnosis before prolonged empirical treatment for a suspected mediastinal lymphoma (21, 25, 39). Where the necessary step is unavailable, the initial impression cannot be corrected, and a cognitive error becomes a system failure.

These barriers are more pronounced in low-resource or conflict-affected settings, where limited imaging capacity and restricted referral or specialist access can prolong time to definitive diagnosis (7, 9). Figure 1 summarizes these interacting clinical, cognitive, and system-level factors.

**Figure 1:**
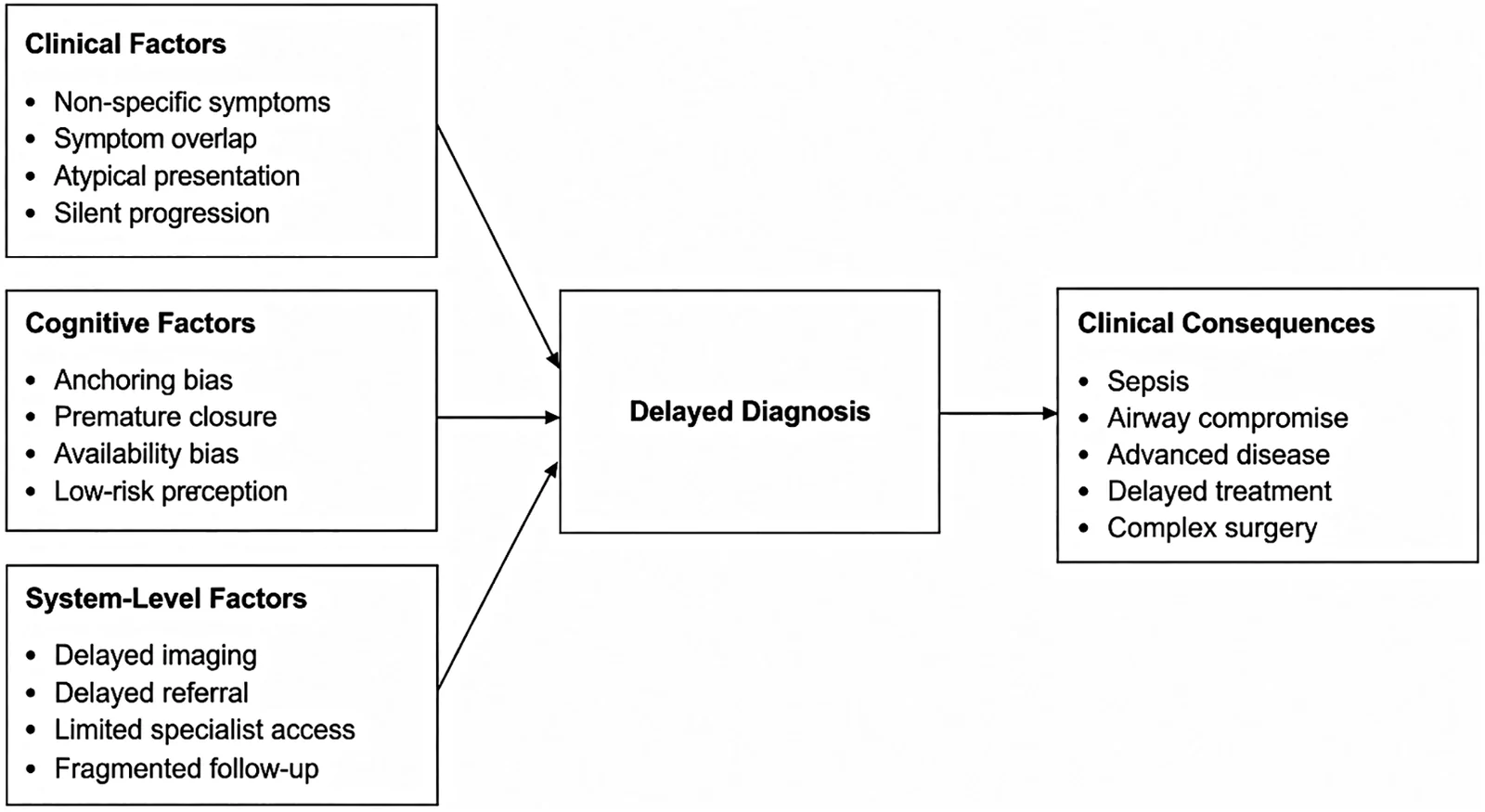
How clinical, cognitive, and system-level factors interact to delay the diagnosis of thoracic and mediastinal conditions in adults aged 18–40. Three domains contribute to delay and reinforce one another. Clinical factors are the non-specific presenting features, including chest or epigastric pain, dyspnea, cough, wheeze, gastrointestinal symptoms, and B symptoms, that overlap with far commoner benign conditions in this age group. Cognitive factors act on that ambiguity: age-based underestimation of risk makes a benign explanation the default, anchoring fixes the initial label, availability bias favors the familiar diagnosis, and premature closure ends the diagnostic process before serious alternatives are excluded. System-level factors determine whether that initial impression is ever corrected, through access to cross-sectional imaging, bronchoscopy, tumor markers, biopsy, specialist referral, and reliable follow-up. The mechanism common to all five conditions reviewed is that when the initial diagnosis is treated and no reassessment is triggered, the error is sustained rather than detected, and each subsequent encounter inherits the original label. The consequences of delay fall into two distinct groups (see Section 4.5): in acute mechanical and perforation emergencies, delay measured in hours is associated with contamination, sepsis, clinical deterioration, and greater operative complexity; in indolent mediastinal disease, delay measured in weeks to months affects diagnostic complexity, symptom and psychological burden, and time to staging and treatment, while prognosis remains strongly influenced by histological subtype and tumor biology. Arrows denote contribution and reinforcement between domains as described in the reviewed literature; they do not represent quantified or causally established pathways.

### 4.5 Contrasting consequences of delay: acute mechanical emergencies versus indolent mediastinal disease

Diagnostic delay is often discussed as though it were a single phenomenon with a single kind of consequence. Across the conditions reviewed here it is not, and conflating the two patterns risks both complacency about the first and overstatement about the second. In acute mechanical and perforation emergencies, esophageal perforation and, in its acute presentation, incarcerated or strangulated diaphragmatic hernia, the relevant unit of delay is hours. Delay in this group operates through a concrete physical mechanism: ongoing mediastinal or peritoneal contamination, progressive tissue edema and friability, evolving sepsis, and, in the case of a strangulated hernia, ischemia of herniated viscera. These processes change what the surgeon encounters, narrowing reconstructive options and shifting management from primary repair towards more complex or staged procedures, and they are associated with worse outcomes. In esophageal perforation this relationship is supported by cohort data and by individual-patient-data meta-analysis in broad adult populations, in which diagnosis after 24 h was associated with substantially higher mortality (3, 26). The direction and mechanism of this association are well supported; its magnitude in adults aged 18–40 specifically has not been established, and the figures reported in older cohorts should not be transferred to younger patients as if they were age-specific estimates. For delayed diaphragmatic hernia the supporting evidence is weaker still, consisting of case series describing emergency presentations with obstruction or strangulation, which establish the clinical pattern but not its frequency or its outcome relative to earlier detection (1, 13, 14).

In PMGCT and HL the currency of delay is weeks to months, and the consequences are of a different kind. Delay postpones tissue diagnosis, staging, and the start of treatment; it prolongs symptom burden and the period of diagnostic uncertainty, which carries its own psychological cost; and, in HL, it is associated with more advanced stage at diagnosis in at least one cohort (25). What the available evidence does not establish is a clear effect of delay on survival in these conditions. Norum found no significant association between delay and stage distribution, relapse rate, or short-term survival in a small retrospective series (23), and Antel et al. likewise found no association between diagnostic delay and overall survival, while noting that delay was associated with late-stage disease and that late-stage disease is itself prognostically adverse (25). These findings are not contradictory, but they are not equivalent to a demonstrated survival benefit from earlier diagnosis either, and they come from small or mixed-age cohorts. Prognosis in both conditions is strongly influenced by histological subtype and tumor biology, non-seminomatous versus seminomatous histology in PMGCT, and histological subtype and stage in HL, and these factors may plausibly account for part of the observed relationship between delay and stage, since more indolent tumors may both present later and behave better (23, 32, 33).

The practical consequence is that the argument for urgency differs between the two groups. In the acute group, urgency is justified by a mechanistic and reasonably well-evidenced link between elapsed time and surgical and clinical outcome. In the indolent group, the case for timely diagnosis rests on avoiding prolonged untreated symptoms, avoiding harmful or unnecessary empirical treatment given for the wrong disease, reducing the psychological burden of prolonged uncertainty, and avoiding presentation with mass-effect complications such as superior vena cava obstruction or airway compression, not on a demonstrated survival gain. Stating this explicitly is more useful to clinicians than implying a uniform prognostic imperative that the evidence does not support, and it is the framing used consistently in Table 1 and in Section 5.

### 4.6 Integrated summary and implications for primary and first-contact care

Overall, delayed diagnosis in these conditions reflects interaction between clinical presentation, cognitive bias, and system-level barriers: non-specific symptoms resembling benign conditions, clinician anchoring on common explanations in patients perceived as low risk, and limited access to imaging, bronchoscopy, tumor markers, biopsy, or specialist follow-up that delays correction of the initial impression.

This synthesis shows why a purely disease-by-disease approach is insufficient. Although each condition has specific diagnostic clues, the shared pattern is clear: persistent, recurrent, progressive, focal, or treatment-resistant thoracic symptoms in a young adult should trigger reassessment of the initial diagnosis rather than continued treatment of it.

Most of these patients are seen first in primary care or another outpatient setting, and it is worth stating plainly what that clinician is working without. Several widely used suspected-cancer referral pathways are explicitly age-thresholded: the NICE suspected-cancer guideline, for example, frames its lung-cancer recommendations, including urgent referral for unexplained hemoptysis and urgent chest radiography for combinations of unexplained cough, fatigue, dyspnea, chest pain, weight loss, and appetite loss, for people aged 40 and over (41). Those thresholds are appropriate to the underlying epidemiology, since the common thoracic malignancies are far rarer below 40. But their effect is that a clinician assessing a 26-year-old with the same symptoms receives no pathway prompt at all, at precisely the moment when the age-based reassurance is doing its most work. Recognition in this age group therefore depends disproportionately on symptom pattern and on the clinician's own reassessment discipline rather than on an external trigger.

Table 2 consolidates the resulting red flags and reassessment points that recur across Sections 3.1–3.5. It is intended as a prompt for reconsideration, not as a screening protocol or a set of validated decision thresholds; the recommendations it contains are proportional to the evidence described in Section 3, and no universal timing rule is proposed.

### 4.7 Limitations

This review has several limitations. As a narrative review, study selection was based on relevance and clinical importance rather than on a formal systematic search, and no formal quality appraisal of the included literature was undertaken by any instrument. Selection and interpretation therefore reflect the authors' judgment, and relevant literature may have been missed; the synthesis should be read accordingly. The included literature varied widely in design, population, and definition of diagnostic delay, which limits comparison across conditions: “delay” denotes elapsed hours in Section 3.1, a healthcare practitioner interval measured in weeks in Section 3.5, and an interval of months to years between injury and presentation in Section 3.2. These are not commensurable quantities and are not treated as such.

The most substantial limitation concerns age. Although the review is framed around adults aged 18–40, most of the foundational evidence on diagnostic delay derives from broader or older adult populations: the cohort evidence linking delay to mortality in esophageal perforation has a median age in the seventh decade, the diagnostic-accuracy evidence in diaphragmatic injury comes from general trauma populations that are not age-stratified, the delay estimates in airway tumors come from adult referral series in a disease predominantly affecting older patients, and the principal HL delay cohort has a median age of 48 years. Only PMGCT is intrinsically concentrated in this age band, and the adolescent and young adult HL literature is age-defined but concerns outcomes and care delivery rather than delay. The “young adult” framing of this review therefore rests substantially on extrapolation and on mechanistic reasoning about how age influences clinical suspicion, rather than on age-specific delay data. We have signposted this distinction wherever evidence is discussed and in Table 1 rather than confining it to this section, but it remains a genuine constraint on the strength of the review's conclusions.

The Hodgkin lymphoma and airway tumor sections rely substantially on case reports, small observational studies, and referral-center data, which limits conclusions regarding the frequency, generalizability, and outcomes of diagnostic delay in these contexts. Finally, several sources describe practice in specific health systems, including a tuberculosis- and HIV-endemic setting, and their transferability to other settings is uncertain.

### 4.8 Implications for practice and research

Reducing diagnostic delay in young adults requires both disease-specific awareness and general diagnostic-safety strategies. Awareness alone is a weak intervention, because the biases described in Section 4.3 operate precisely when the clinician believes the diagnosis is settled. Croskerry proposed cognitive forcing strategies as a response to this problem: structured, deliberately triggered prompts that interrupt automatic reasoning at a predefined point, applied at universal, generic, and disease-specific levels, with the specific level directed at recognized pitfalls in clinical reasoning (42). Wright et al. similarly identified structured checklists, improved handover, and cognitive-bias education as interventions with supporting evidence in emergency settings (9). Applied to the conditions reviewed here, four checkpoints follow directly from the mechanisms described in Section 3, and are triggered by an event rather than by the elapse of time alone.

First, a diagnostic time-out when an expected treatment response does not occur. Where a patient treated for asthma has not obtained the expected benefit, the operative question is not what to add but whether the diagnosis is right. Current primary-care asthma guidance supports reviewing the diagnosis and considering alternative causes in this situation, and uses a trial of approximately four weeks of anti-inflammatory treatment as one benchmark for confirming the diagnosis (38). We propose this as a structured review point, not as a mandatory rule: no evidence identified establishes a specific interval after which airway pathology must be excluded, and a checkpoint that fires on absence of expected response is both better supported and more clinically sensible than one that fires on the calendar alone.

Second, a pause before empirical anti-infective treatment substitutes for tissue diagnosis. Where a mediastinal or nodal mass with B symptoms is treated empirically for tuberculosis without microbiological confirmation, the checkpoint is to establish in advance what result would prompt biopsy and by when, rather than to review at an unspecified future point. Empirical tuberculosis therapy was associated with a longer time to lymphoma diagnosis in the cohort of Antel et al., and non-diagnostic fine-needle aspiration was itself associated with further delay (25).

Third, completion of the diagnostic dataset before treating an anterior mediastinal mass. In a young man, AFP, *β*-HCG, and LDH should be obtained before treatment is directed at a presumed alternative diagnosis (33, 35, 36). We describe this as a structured expectation rather than a mandatory panel, since no identified authoritative source frames marker testing as an obligatory precondition of treatment.

Fourth, reconsideration of occult diaphragmatic injury when unexplained symptoms follow thoracoabdominal trauma. A previous thoracoabdominal injury, however remote and however thoroughly assessed at the time, should be treated as an active item in the differential when later unexplained gastrointestinal or thoracic symptoms develop (1, 13, 14, 31). These are proposals derived from the reviewed literature and from established diagnostic-safety theory. None has been prospectively evaluated in this population, and we do not present them as validated interventions.

Future research should prioritize prospective studies specifically in adults aged 18–40, including in low-resource and conflict-affected settings, since the evidence gap identified in Section 4.7 is the principal constraint on this field. Priorities include age-stratified reporting of existing delay cohorts, which would require no new data collection; quantification of the diagnostic interval in conditions where it is currently described only in case reports; and evaluation of whether structured checkpoints of the kind proposed above reduce delay without generating unacceptable rates of low-yield imaging in a population where the prior probability of serious disease is genuinely low. That trade-off, rather than awareness alone, is the substantive question for future work.

## 5 Conclusion

Delayed diagnosis of selected thoracic and mediastinal conditions in young adults is not attributable simply to the rarity of these diseases; it arises from the combined effects of clinical, cognitive, and systemic barriers (3, 5–7, 13, 17). Non-specific symptoms overlapping with benign disease make early diagnosis difficult, and this difficulty is amplified by the perception of young adults as being at low risk of serious thoracic pathology, by anchoring, premature closure, and availability bias, and by system-level constraints on imaging, specialist referral, and tissue diagnosis (5–7, 9, 10).

The consequences of delay differ by condition and should not be described uniformly. In acute mechanical and perforation emergencies, delay may be measured in hours and is associated with greater contamination, clinical deterioration, and operative complexity. In indolent mediastinal malignancies, delay principally affects diagnostic complexity, symptom and psychological burden, and time to staging and treatment, while prognosis remains strongly influenced by histological subtype and tumor biology; the prognostic effect of delay in Hodgkin lymphoma in particular remains uncertain (23, 25).

For the clinician, the practical message is a reassessment discipline rather than a list of rare diagnoses to memorize: thoracic symptoms in a young adult that are focal, persistent, progressive, recurrent in the same distribution, or unresponsive to appropriate treatment warrant reconsideration of the original diagnosis, with the disease-specific next steps summarized in Table 2. This is particularly important below the age of 40, where age-thresholded referral pathways provide no external prompt (41). Because the delay literature in this age group remains largely extrapolated from broader adult populations, prospective, age-stratified study of diagnostic delay in young adults, including in settings where imaging, specialist availability, and referral infrastructure are constrained, is the necessary next step (7, 9).
